# Supplementary figures and images for: Evolution of Reproductive Life History in Mammals and the Associated Change of Functional Constraints
Source: Genes (Basel). 2021 May 14;12(5):740. doi: 10.3390/genes12050740 (PMC8157036; doi:10.3390/genes12050740)

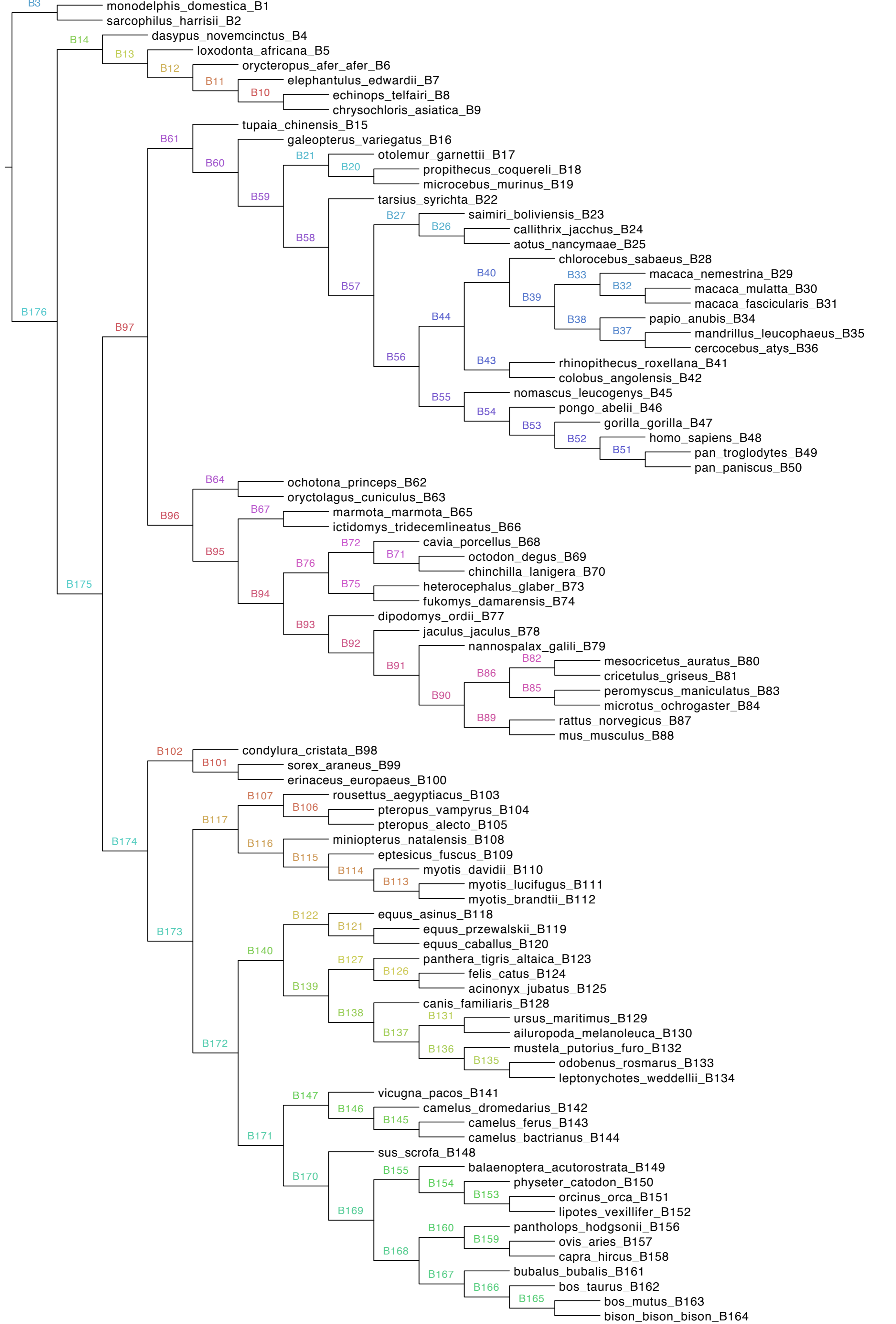

Supplement: Supplementary file 1 [file genes-12-00740-s001.zip › SupplementalTables/FigureS1.annotation89mam.pdf]
